# Using a generative model of affect to characterize affective variability and its response to treatment in bipolar disorder

**DOI:** 10.1073/pnas.2202983119

**Published:** 2022-07-05

**Authors:** Erdem Pulcu, Kate E. A. Saunders, Catherine J. Harmer, Paul J. Harrison, Guy M. Goodwin, John R. Geddes, Michael Browning

**Affiliations:** ^a^Department of Psychiatry, University of Oxford, Oxford, OX3 7JX, United Kingdom;; ^b^Oxford Health National Health Service Trust, Warneford Hospital, Oxford, OX3 7JX, United Kingdom

**Keywords:** affect, bipolar disorder, lithium, computational modeling

## Abstract

The affective variability of bipolar disorder (BD) is thought to qualitatively differ from that of borderline personality disorder (BPD), with changes in affect persisting longer in BD. However, quantitative studies have not been able to confirm this distinction. It has therefore not been possible to accurately quantify how treatments like lithium influence affective variability in BD. We assessed the affective variability associated with BD and BPD as well as the effect of lithium using a computational model that defines two subtypes of variability: affective changes that persist (volatility) and changes that do not (noise). We hypothesized that affective volatility would be raised in the BD group, noise would be raised in the BPD group, and that lithium would impact affective volatility. Daily affect ratings were prospectively collected for up to 3 y from patients with BD or BPD and nonclinical controls. In a separate experimental medicine study, patients with BD were randomized to receive lithium or placebo, with affect ratings collected from week −2 to +4. We found a diagnostically specific pattern of affective variability. Affective volatility was raised in patients with BD, whereas affective noise was raised in patients with BPD. Rather than suppressing affective variability, lithium increased the volatility of positive affect in both studies. These results provide a quantitative measure of the affective variability associated with BD and BPD. They suggest a mechanism of action for lithium, whereby periods of persistently low or high affect are avoided by increasing the volatility of affective responses.

## Significance

Extreme mood variability causes significant difficulties in bipolar disorder (BD) and borderline personality disorder (BPD). It is not clear how to conceptualize or measure mood variability, which makes it challenging to assess how treatments for these conditions work. We developed and deployed a computational model, which estimates whether mood variation is persistent versus transient, in patients with BD and BPD, and in a randomized study of lithium. We found that the BD group displayed persistent changes in mood, whereas the BPD group experienced transient changes. Lithium increased persistent changes of positive mood. This work demonstrates that specific types of mood variability are associated with BD and BPD, and suggests a way of understanding how lithium prevents extreme mood states.

Excessive affective variability, sometimes called affective instability, characterizes psychiatric diagnoses such as bipolar disorder (BD) and borderline personality disorder (BPD) (1–4), and is associated with adverse outcomes across diagnoses (5, 6). It has been suggested that affective instability may be an important treatment target across a range of psychiatric presentations (3, 7, 8).

Different types of affective variability are thought to exist; when asked to retrospectively describe their experiences, patients with BD report longer periods of raised or lowered affect, whereas patients with BPD report a higher frequency variation of affect (4). Consistent with this difference, mood stabilizing medications such as lithium, which reduce the occurrence of mania and depression (i.e., particularly prolonged periods of extreme affect) in BD (9), have not been found to be effective in patients diagnosed with BPD (10).

Affective variability may be directly estimated from prospectively collected affect ratings, with a variety of different metrics of variability described (11–14). However, the different measures of variability tend to be highly correlated with one another (14) and to date do not clearly capture the qualitative differences in duration of affective changes described by patients. That is, previous work with prospectively collected data has not shown longer-lasting changes of affect in BD and shorter-lived changes in BPD. Rather, the same measures of affective variability that are raised in BPD (11, 15–18) are generally also raised, to a somewhat lesser degree, in bipolar disorder (11), posttraumatic stress disorder, and bulimia nervosa (16). Existing measures of variability of affect ratings therefore lack diagnostic specificity and cannot account for differences in treatment response between diagnoses.

**Fig. 1.**
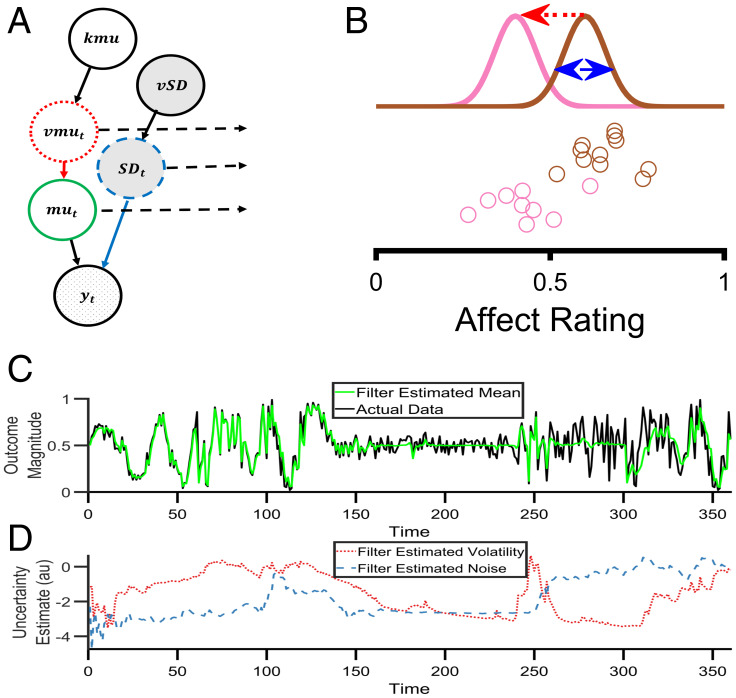
A Bayesian filter to estimate types of affective variability. (*A*) A graphical illustration of the generative model that describes how affect ratings (represented by $y_{t}$) are produced at each time point. The hypothesized causal processes leading to the production of the ratings is controlled by the nodes $mu_{t}$, $SD_{t}$, $vmu_{t}$,kmu, and vSD, which are described in the main text. (*B*) An illustration of the types of variability in the generative model. Circles represent individual affect ratings, sequentially generated from top to bottom. The color of the circle indicates the distribution from which it was drawn. One type of variability, volatility ($vmu_{t}$, red arrow), arises from a shift in the distribution (from brown to pink), leading to a change in all subsequent ratings. A second type of variability, noise ($SD_{t}$, blue arrow), arises from the sampling of the ratings from the distributions and leads to independent changes in each rating. (*C*) Behavior of the Bayesian filter using synthetic data. The black line illustrates a time series of synthetic data drawn from the range 0–1. The data contains periods in which volatility is high (time 1–120 and 301–360) and others in which it is low (time 121–300). Similarly, it contains periods in which noise is high (time points 61–120 and 241–360) and low (time 1–60 and 121–240). The green line illustrates the Bayesian filter's belief about the mean of the generative process, $mu_{t}$, at each time point. As can be seen, the filter changes its estimate of the mean when it thinks variability in the data is caused by volatility (e.g., time 1–60) and does not alter its estimate of the mean when it thinks variability is caused by noise (e.g., time 260–300). It is able to adapt to changes in the level of volatility and noise, although it occasionally misattributes the cause (e.g., when the noise increases at time 240, the filter initially believes this is caused by an increase in volatility before correctly attributing it to noise by time 260). (*D*) The filter's estimate of volatility (red line) and noise (blue line) from the same synthetic data as (*C*). Panels *C* and *D* are adapted from ref. 20.

An alternative approach to conceptualizing and measuring the variability of an outcome is to construct a generative model of how that outcome is produced and then to invert the model using Bayes’ rule (19, 20). A generative model formally describes the assumed causal processes that produce an outcome (Fig. 1); inversion of the model creates a “Bayesian filter” (19–22), which allows one to start with the observations and then to estimate distinct, model-defined causes of variability within a single, overarching framework.

In this paper, we inverted a simple generative model of affect, as measured using ratings of momentary affect (Fig. 1), to estimate two different causes of affective variability, captured as changes in the affective ratings over time: volatility, which leads to persistent change in affect, and noise, which leads to transient change. We applied this approach to prospectively collected affect ratings of patients with BD and BPD as well as control subjects to assess whether it was able to capture the qualitative differences in affective variability between these diagnostic groups. We then used the model to characterize the causal effects of lithium on affective variability in an experimental medicine study of patients with BD. We hypothesized that BD would be associated with increased affective volatility and BPD with increased affective noise, and that lithium would impact affective volatility.

## Results

The generative model of affect and associated Bayesian filter are summarized in Fig. 1. A detailed description and assessment of the performance of the filter is provided in the *Materials and Methods* section and the *SI Appendix*. The key feature of the filter is that it estimates two forms of variability: that caused by affective volatility (i.e., changes of affect that persist over time) and that caused by affective noise (i.e., changes in affect that are transient). We first used the filter to characterize prospectively collected, daily, positive, and negative affect ratings (11) from a cohort study of patients with diagnoses of BD (*n* = 53), BPD (*n* = 33) and nonclinical controls (*n* = 53), see *SI Appendix*, Table S1.

### Distinct Types of Affective Variability in BD and BPD.

**Fig. 2.**
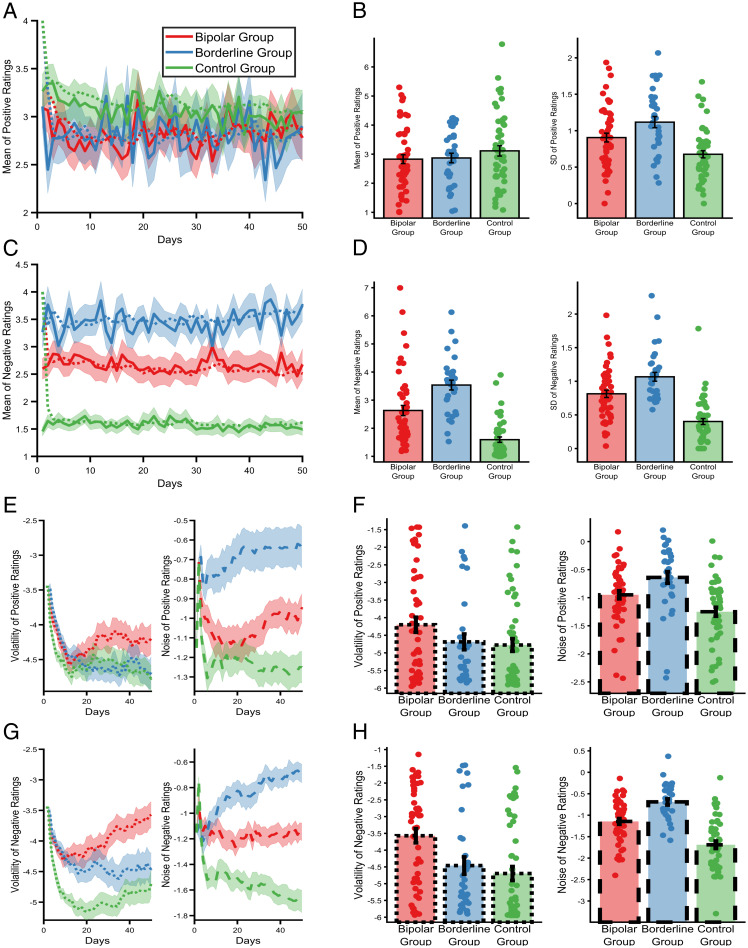
The types of affective variability in people with a diagnosis of bipolar disorder (BD), borderline personality disorder (BPD), or no diagnosis. Mean positive (*A*) and negative (*C*) daily affect ratings across 50 d of the study (solid lines illustrate means, ± SEMs are represented by shaded regions). The predicted mean scores of the Bayesian filter (i.e., the expected values of $mu_{t}$ before observing that day's rating) are superimposed as dashed lines. Summary statistics (mean and SD) of positive (*B*) and negative (*D*) affect ratings calculated across the 50 d. As can be seen, positive affect ratings did not differ between groups, whereas ratings of negative affect differed significantly, with the BPD group reporting the highest scores, followed by the BD group and then the control group. The same ordering of groups was found for affective variability of both positive and negative affect as estimated by the SD of the ratings (11). Evolution of the Bayesian filter's beliefs about the causes of positive (*E*) and negative (*G*) affective variability across the same 50 d of the study. Lines represent the means (± SEMs) of the expected values of the $vmu_{t}$ node, for volatility, and $SD_{t}$ node, for noise. The filter’s estimates change quickly when it is first presented with data, stabilizing after ∼10–20 d. However, the filter is able to detect later changes in the parameters, resulting in ongoing fluctuations of estimated parameters. Final beliefs of the Bayesian filter (i.e., at day 50) about the types of variability for positive (*F*) and negative (*H*) affect. The filter attributes different types of affective variability to the two clinical groups, with noise being higher in the BPD group and volatility in the BD group. For all panels, data from the BD group is summarized in red, the BPD group in blue, and the control group in green lines or bars. Lines and bars reporting volatility are dotted, those reporting noise are dashed, and those reporting other measures (means, SDs) are solid.

When considering the standard summary statistics (14) of the affective ratings from the three cohorts, the average ratings of positive affect did not differ between groups [*F*(2,132) = 1.52, *P* = 0.26], although negative ratings did differ (*F*(2,132) = 32.26, *P* < 0.001), with patients in the BPD group endorsing higher mean ratings than both of the other groups (both *p_bonf_*s < 0.006), and patients in the BD group providing higher ratings than the control group [*p_bonf_* < 0.001]. An identical ordering of the groups was apparent for both positive (*F*(2,122) = 11.6, *P* < 0.001) and negative (*F*(2,122) = 38.2, *P* < 0.001) affective variability, as estimated using the SD of the ratings (Fig. 2 *A–D*) and, as previously reported, other measures of variability, including the RMS (root mean square) of successive differences, the entropy, and the Teager-Kaiser energy operator (11). Thus, although the magnitude of the variability metrics differed between groups, there was no specific association between qualitative types of variability and diagnosis, with all of the measures being higher in the BPD group than the BD group.

Applying the Bayesian filter to these data (Fig. 2 *E–H*) provided clear evidence of a specific association between distinct types of affective variability and diagnosis (group × type of variability; *F*(2,122) = 7.92, *P* = 0.001), which did not differ between positive and negative ratings (group × cause of variability × valence; *F*(2,122) = 1.91, *P* = 0.15). As can be seen, across both positive and negative ratings, estimated volatility was higher in the bipolar group than in both the borderline (*p_bonf_* = 0.042) and control (*p_bonf_* < 0.01) groups, with the difference between the borderline and control groups being nonsignificant (*p_bonf_* = 0.6). In contrast, estimated noise was higher in the BPD group than in both the BD (*p_bonf_* = 0.016) and control (*p_bonf_* < 0.001) groups and was also higher in the BD than in the control (*p_bonf_* < 0.001) group. In other words, the filter-estimated types of affective variability are diagnostically specific, with volatility being higher in patients with BD and noise higher in patients with BPD.

### Ongoing Lithium Treatment Is Associated with Increased Volatility of Positive Affect.

**Fig. 3.**
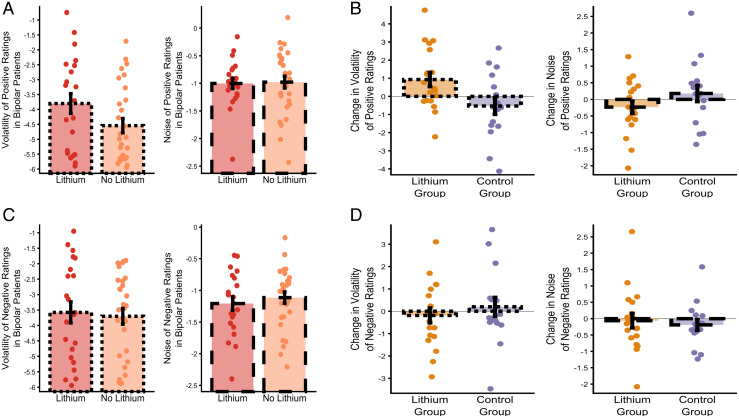
Lithium specifically increases the volatility of positive affect in patients with BD. The volatility and noise of positive (*A*) and negative (*C*) affective ratings in patients from the BD group of the cohort study who were and were not receiving treatment with lithium are shown. The charts illustrate the mean (± SEM) of volatility and noise at day 50. Patients receiving lithium have a higher volatility of positive affect (panel *A*), with no effect on the noise of positive ratings or on either outcome for negative ratings (panels *A* and *C*). Although this result raises the possibility that lithium causes an increase in positive affective volatility, strong evidence for causality requires a randomized design. Panels *B* and *D* illustrate the results of a randomized trial of lithium, with the change in the volatility and noise of positive (*B*) and negative (*D*) affective ratings across the treatment period of the study shown. The charts illustrate the mean (± SEM) of the changes in volatility and noise at day 28 (relative to the end of the run-in period). As can be seen, the results of this randomized study are consistent with those from the cohort study, with lithium producing a specific increase in the volatility of positive affect ratings, with no effect on the other measures. Data from participants in the bipolar group of the cohort study who are being treated with lithium are summarized in red and those not taking lithium by orange bars. From the experimental medicine study, participants randomized to lithium are represented by yellow/orange and those to placebo by purple bars. Bars reporting volatility have dotted edges and those reporting noise have dashed edges.

Of the 51 patients with a diagnosis of BD recruited to the cohort study, 22 were receiving ongoing lithium treatment and 29 were not. As illustrated in Fig. 3 *A* and *C*, the volatility of positive ratings was raised in patients with a diagnosis of BD who were receiving lithium treatment compared to those who were not (*F*(1,41) = 6.27, *P* = 0.023), with no difference in any of the other filter-derived metrics (all *F*s < 0.019, *p*s > 0.89) and no difference in the mean or SD of the ratings (all *F*s < 1.425, *p*s > 0.51). Including lithium treatment as a factor in the analysis of the volatility data from the cohort study indicated that levels of positive volatility did not differ between the groups (main effect of group *F*(2,121) = 0.35, *P* = 0.71), but were influenced by lithium treatment status (main effect of lithium *F*(1,121) = 4.66, *P* = 0.033). In contrast, negative volatility differed between the groups (main effect of group *F*(2,121) = 7.14, *P* = 0.001) and was not influenced by lithium (main effect of lithium *F*(1,121) = 0.04, *P* = 0.84). These results raise the possibility that lithium treatment increases the volatility of positive affect, although the design of this longitudinal study does not permit firm conclusions as to the causal effect of lithium treatment.

### Initiation of Lithium Specifically Increases Positive Affective Volatility in BD.

The causal influence of lithium on affective variability was therefore assessed using data from the Oxford Lithium (OxLith) trial (23). In this study, patients with a diagnosis of BD were randomly assigned to 4-wk treatment with lithium or placebo, with daily affect ratings completed from 2 wk before treatment initiation to 4 wk after. Lithium treatment had no effect on the means (all interactions including treatment; *F*s (1,33) < 1.6, *p*s > 0.21) or SDs (*F*s (1,33) < 0.67, *p*s > 0.42) of either the positive or negative affect ratings. However, lithium differentially altered affective variability, estimated using the Bayesian filter, as a function of both its type and valence (treatment × type of variability × valence *F*(1,33) = 5.68, *P* = 0.02). As can be seen in Fig. 3 *B* and *D* and consistent with the results from the cohort study (Fig. 3 *A* and *C*), lithium specifically increased the volatility of positive affect ratings (effect of treatment *t*(33) = 2.17, *P* = 0.04), without altering negative volatility (*t*(33) = −0.9, *P* = 0.39) or the noise of either valence (*t*s(33) < 1.36, *p*s > 0.18).

## Discussion

Patients with BD and BPD were found to have distinct types of affective variability, as defined by a generative model of affect. Affective volatility was increased in patients with BD, whereas affective noise was increased in patients with BPD. Treatment with lithium specifically elevated the volatility of positive affect.

There has been debate about the types of affective variability that may exist, how the different types should be defined, and whether they add to simpler metrics such as the mean and SD of affective ratings (11–14). The measures derived from our model indicate diagnostic specificity related to the duration of affective changes between patients with diagnoses of BD and BPD. This observed association, with increased affective volatility in patients with BD and increased affective noise in patients with BPD is consistent with the qualitative descriptions of the disorders (4) and indicates that the model formally captures clinically relevant aspects of affective variability that are not apparent using simpler metrics (2, 11, 15).

The Bayesian filter estimates different forms of variability within a single framework. A range of alternative measures of affective variability have previously been described (14). Of these, the measure most closely linked to affective volatility is affective inertia, often formalized as the slope of a first-degree autoregressive (AR1) model (24, 25), with affective noise being similar to the SD of the residuals of that model. Previous work has associated increased affective inertia with reduced functioning (24, 25) and analysis of the current data using an AR1 model produced a similar overall pattern of results for the cohort study, although it did not replicate the difference between the bipolar and borderline clinical groups found for volatility (see *SI Appendix*).

We found that lithium, an agent with proven efficacy for treating and averting extreme affective states in BD (9, 26), specifically increased the volatility of positive affect of patients with BD in both a real-world cohort study and a randomized experimental medicine study. A question raised by these results is how an increase in positive affective volatility may relate to the clinical effects of lithium, particularly its ability to terminate or avoid extreme mood states (26). One explanation relates to a characteristic feature of mania and depression—that, during an episode, patients’ affect becomes stuck at an extreme. Affective volatility is a change in affect that persists across time, suggesting that increased positive volatility may be exactly what is required to escape the affective confines of manic or depressed episodes. In other words, lithium does not act to simply suppress affective variability, as may be assumed of a “mood stabilizing” treatment, but rather to enhance a particular type of affective variability that can prevent patients becoming stuck in periods of mania or depression. This interpretation raises a number of questions for future study, most obviously whether the clinical impact of lithium is related to its effect on positive volatility and whether other interventions that target affective noise and negative volatility may be identified.

The Bayesian filter attributes changes in affect that persist across sampling points to volatility, whereas changes that do not persist are attributed to noise (see *SI Appendix, Methods*). This suggests an interpretation of the present results in terms of the “half-life” of affective responses: Patients with BD have a longer half-life of affective response than patients with BPD (at least for negative affect) and lithium acts to increase the half-life of positive affective responses. This formulation is consistent with previous qualitative descriptions of these patient groups that have highlighted the shorter time scale of affective responses in patients with a diagnosis of BPD (27) and suggests that the filter-derived metrics may provide a particularly useful quantitative assessment of patients who lie at the diagnostic boundary, such as those with rapidly cycling BD (27). More broadly, other characteristics of the affective response to provoking events influence the filter-derived metrics. In particular, an increase in the magnitude or frequency (see *SI Appendix, Materials*) of affective response results in higher levels of both volatility and noise. This suggests that both patient groups either experience more provoking events, or react more readily or to a greater degree to such events, than control participants. In the present study we did not measure the occurrence of provoking events and therefore cannot directly test this proposal, although it would amenable to future work incorporating measures of stressful life events.

An important limitation of this work relates to the characteristics of the cohorts used in the first study. In this study, the control group was specifically matched to the BD, rather than the BPD group. As a result, patients in the BPD group had lower educational achievement and were much less likely to be male. Although the same pattern of results was found when analyzing only female patients (see *SI Appendix, Results*), and these demographic variables were included in the analyses, it would clearly be desirable to replicate the present findings in separate cohorts in which these demographic factors are more closely matched. There are important differences between the cohort and experimental medicine studies. In the latter, patients were earlier in their presentation to services and not receiving treatment, whereas patients recruited to the cohort study were largely receiving established treatment. Furthermore, the two studies differed in the rating scales used to estimate affect. Although these differences limit the degree to which the studies are directly comparable, the similarity in the apparent effect of lithium across the studies suggests that this effect is not dependent on these factors.

Computational psychiatry uses formal descriptions of mechanistic processes to better understand psychiatric illness and enhance the development of treatments (28). Taking this approach, we have deployed a generative model and associated Bayesian filter to describe and measure distinct types of affective variability in patients with BD and BPD, and have found that lithium acts to specifically increase positive affective volatility.

## Materials and Methods

Details of the cohort and experimental medicine studies are provided in sequential sections.

### Cohort Study.

#### Overview.

The Automated Monitoring of Symptom Severity (AMoSS) study recruited cohorts of patients diagnosed with BD and BPD, as well as nonclinical participants, to examine the relationship between affect, activity, and physiological measures. Results from the AMoSS study, including summary measures of affect ratings and validation of the ratings used have been previously reported by Tsanas and colleagues (11). All of the participants gave written informed consent to participate in the study, which was approved by the East of England, Norfolk National Health Service (NHS) ethics committee (13/EE/0288). Participants were asked to complete 3 months of daily ratings of positive and negative affect, with the option to continue indefinitely beyond this point. Participants were also asked to provide demographic data, including age, sex, and educational attainment.

#### Participants.

Patients were recruited from services in Oxfordshire and from the local community. Control participants were sex and age matched to patients from the bipolar group. All of the participants were assessed by a consultant psychiatrist who confirmed diagnoses of bipolar disorder using the structured clinical interview for the *Diagnostic and Statistical Manual of Mental Disorders, Fourth Edition* (*DSM-IV*) (SCID-IV) (29) and of BPD using the appropriate section of the International Personality Disorder Examination (IPDE) (30). Control participants were screened using the SCID to confirm no current or previous diagnosis.

A total of 53 patients diagnosed with BD, 33 diagnosed with BPD, and 53 control participants were recruited to the study. One participant withdrew consent. For the current analysis, participants were included if they had completed at least 10 affect ratings (as estimates of volatility stabilized at this point, see Fig. 2). This left 51 patients with BD, 33 patients with BPD, and 51 controls. As the control group was selected to match the BD group, the group of patients with BPD differed from the other two groups with a higher proportion of female patients and lower average educational attainment. These variables were included as covariates in the reported statistics (and analysis restricted only to female participants produced the same group × type of affective variability effect; see *SI Appendix, Materials*).

#### Measure of affect.

Participants completed daily affect ratings using the “moodzoom” Android app (participants without an Android phone were supplied with one for the duration of the study). The moodzoom app prompted participants to rate their current affect every evening by endorsing each of six descriptors (anxious, elated, sad, angry, irritable, energetic) on a 7-point Likert scale. Summary positive and negative affect scores were calculated as the average of the positive and negative items (11). The 50-d period for each participant that had the fewest missing data points was used for analysis (identical group effects were observed if the first 50 ratings for each participant were used).

### Experimental Medicine Study.

#### Overview.

The OxLith trial was a randomized, controlled experimental medicine study of patients with bipolar spectrum disorder, conducted in Oxford, UK (23). During an initial screening visit, diagnosis was confirmed using the SCID-IV. Participants then completed a 2-wk, prerandomization, run-in period, after which they were randomized to receive lithium carbonate or placebo for up to 6 wk. Participants completed daily ratings of positive and negative affect throughout the run-in and postrandomization periods. All of the participants provided written informed consent to participate in the study, which had been approved by the NHS South Central Research Ethics Committee (15/SC/0109). The study protocol was registered (ISRCTN91624955) and published (23) before study completion. The analysis reported in the present paper is an additional exploratory analysis not described in the protocol.

#### Participants.

The study recruited individuals aged ≥18 y, with a diagnosis of BD (bipolar I, II, or not otherwise specified [NOS]) for whom there was uncertainty about whether treatment with lithium was appropriate (e.g., an individual with a recent diagnosis of BD or who has experienced relatively few severe mood episodes). Individuals were recruited from local clinical services. Individuals were not eligible for the trial if they had any contraindications to lithium treatment, were taking concomitant psychotropic medication that they were unable to discontinue, had clinically significant substance misuse, required urgent treatment for a mood disorder (i.e., where placebo treatment would be unethical), were pregnant or of childbearing age and not using effective contraception, or were acutely suicidal. Summary demographic data are presented in *SI Appendix*, Table S1.

#### Randomization, intervention, and blinding.

Participants were randomized using a 1:1 allocation scheme, which was minimized for participant age (<25 y, ≥25 y) and sex (female, male). The active group received lithium carbonate 200 mg prolonged release tablets, which were titrated to a target serum level of 0.7 mmol/L as per routine practice. The trial psychiatrist and participants remained blind to treatment allocation. For participants in the placebo group, sham lithium levels were provided to the treating psychiatrist who then adjusted the placebo “dose.”

#### Measure of affect.

Participants completed an online daily version of the positive and negative affect scale, 10-item version (PANAS) (31). The PANAS requires participants to rate five positive descriptors (alert, inspired, determined, attentive, active) and five negative descriptors (upset, hostile, ashamed, nervous, afraid) on a five-point scale. Summary positive and negative affect ratings were calculated as the average of the positive and negative ratings, respectively.

### The Bayesian Filter.

Here, we provide a summary of the generative model of affect and associated Bayesian filter. A formal description and a comparison with alternative models/measures is provided in the *SI Appendix, Materials*. In the generative model (Fig. 1*A*), one rating per time point, ($y_{t}$), is drawn from a Gaussian probability distribution with a mean, $mu_{t}$, and a SD, $SD_{t}$, (Fig. 1*B*). The mean can change between time points, with this change controlled by the volatility parameter, $vmu_{t}$. Two higher level parameters, kmu and vSD, control the change over time of the volatility and SD, respectively, allowing the model to account for periods during which the volatility and/or SD are high and periods when they are low. The generative model defines two causes of variability of the ratings (Fig. 1*B*) (20): First, a change in the mean of the distribution between trials can cause variability in the ratings (e.g., if the mean has decreased, then the ratings of the next trials will, on average, be lower). The size of this variability is controlled by the volatility parameter, $vmu_{t}$. Second, the production of the ratings from a Gaussian distribution leads to variability about the mean that influences the current rating but has no carryover effects. The size of this variability, which we call noise, is controlled by the SD, $SD_{t}$, of the distribution.

The Bayesian filter inverts this generative model. It starts with the affect ratings, $y_{t}$, and uses these to recursively update its belief about the state of the five generative processes (the circles above $y_{t}$ in Fig. 1*A*) that cause the ratings. As a result, the filter estimates, for each point in time, the degree to which the variability in ratings is produced by volatility and the degree to which it is produced by noise (Fig. 1*D*).

Where more than one set of ratings was provided in a day, the first was used. Days in which no ratings were provided were treated as missing with no data extrapolation (see *SI Appendix, Materials* for an illustration of how the Bayesian filter deals with missing data and for sensitivity analysis of data missingness).

### Statistical Analysis.

Analysis of filter-based data from the cohort study was performed using repeated measures ANOVAs with the within-subject factors of cause of variability (volatility, noise) and valence (positive, negative) and the between-subject factor of group (bipolar group, borderline group, control group). In addition, age, sex, and educational attainment were included as control variables in all of the analyses. In these analyses, the dependent variables were the filter-derived estimates of volatility and noise at day 50. Where post hoc comparisons between the three groups were performed, Bonferroni correction was carried out. These are indicated in the text and report the obtained *P* value multiplied by three to account for the three possible group comparisons. The filter-based data were not normally distributed and so were boxcox transformed (λ = 0.2) before entry into the analysis. As demographic data were missing from some participants (see *SI Appendix*, Table S1), the reported statistical analyses are limited to participants with complete demographic data (omission of these control variables and inclusion of all of the participants in the analysis or analysis of the untransformed data does not alter the significance of results). Data from all of the participants are included in the figures. The nonfilter-based metrics (mean and SD) were analyzed separately (as the mean is not a cause of variability) with a single within-subject variable of valence.

Analysis of data from the experimental medicine study was carried out using a repeated measures ANOVA with the within-subject factors of cause of variability (volatility, noise) and valence (positive, negative) and the between-subject factors of group (lithium, placebo). The dependent variables used were the change in filter-derived estimates of variability between the end of the run-in period and the end of the treatment period. These data did not violate normality assumptions and so were not transformed. All of the inferential statistical tests were two sided. Analyses were performed using IBM SPSS version 25.

## Supplementary Material

Supplementary File

## Data Availability

Some study data are available. The cohort and experimental medicine studies did not obtain consent to upload data onto open platforms; however, anonymous data can be shared with other research groups who have ethical approval in place on a by-project basis. Requests for data access should be made to kate.saunders@psych.ox.ac.uk).
